# A real-time forecasting and estimating system of West Nile virus: a case study of the 2023 WNV outbreak in Colorado, USA

**DOI:** 10.1098/rsos.240513

**Published:** 2024-12-04

**Authors:** Chunlin Yi, Lee W. Cohnstaedt, Caterina M. Scoglio

**Affiliations:** ^1^ Department of Electrical and Computer Engineering, Carl R. Ice College of Engineering, Kansas State University, Manhattan, KS 66506, USA; ^2^ National Bio- and Agro-Defense Facility, Agricultural Research Service, United States Department of Agriculture, Manhattan, KS 66502, USA

**Keywords:** West Nile virus, ensemble Kalman filter, prediction, compartmental models, early warning systems, public health preparedness

## Abstract

West Nile virus (WNV) is a mosquito-borne arbovirus that remains a persistent public health challenge in the USA, with seasonal outbreaks that can lead to severe cases. In this study, we detail a real-time prediction system for WNV that incorporates an adapted compartment model to account for the transmission dynamics among birds, mosquitoes and humans, including asymptomatic cases and the influence of weather factors. Using data assimilation techniques, we generate weekly WNV case forecasts for Colorado in 2023, providing valuable insights for public health planning. Comparative analyses underscore the enhanced forecast accuracy achieved by integrating weather variables into our models.

## 1. Introduction

West Nile virus (WNV) is a mosquito-borne arbovirus first identified in the West Nile District of Uganda in 1937. Since then, WNV has spread to other parts of the world, including Europe, the Middle East, North America and West Asia, and has received widespread attention [1]. The United States has experienced seasonal outbreaks of WNV since its initial detection in New York City in 1999 [2]. Some years have seen a particularly high number of reported cases. For example, in 2003, there were over 9800 reported cases of WNV in the USA [3].

The year 2012 was marked by the highest number of severe cases and deaths, with over 5600 total infections, 2873 neuroinvasive cases and 243 deaths [4]. Recent data from the Centers for Disease Control and Prevention (CDC) [5] indicate that in 2023, there were 2406 detected WNV cases in the USA, with Colorado reporting the highest number of cases (631) among states. As WNV outbreaks occur annually in the USA, long-term predictions of their timing, magnitude and duration are crucial for public health officials in resource allocation and implementing targeted prevention and control measures. Additionally, accurate parameter estimation and short-term real-time prediction of ongoing outbreaks are essential for immediate response and intervention, resource optimization and predictive model refinement.

Recently, forecasting the outbreaks and severity of WNV and other infectious diseases has become a critical area of research. To achieve this, a diverse range of approaches has been developed. These include statistical methods, such as time series analyses [6], logistic regression models [7] and machine-learning approaches [8], that focus on fitting historical observations to identify risk factors and predict outbreak likelihood. Additionally, mechanistic models that utilize the susceptible–infected–recovered (SIR) framework cooperating with data assimilation methods are also employed to estimate and forecast the transmission dynamics of disease outbreaks [9,10]. The CDC’s WNV forecasting challenge is an example of improving public health response to WNV through predictive models. In this challenge, participating teams are provided with annual West Nile neuroinvasive disease (WNND) cases from previous years to forecast annual WNND cases for each county in the contiguous United States using a variety of modelling approaches [8,11,12]. However, year-long forecasting of WNV is inherently challenging due to a variety of factors, including the unpredictability of local weather patterns, ecological changes and vector population dynamics. Numerous studies have underscored how these environmental factors influence WNV transmission [13–15]. This variability presents challenges in accurately capturing the outbreak dynamics in regions experiencing erratic weather conditions. To address this issue, the development of a real-time forecasting system could provide a solution, offering more immediate and adaptive predictions to guide public health interventions.

For the real-time forecasting of infectious disease, a model-inference framework that consists of an epidemiological model, up-to-date surveillance data and a data assimilation method has been widely used [16,17]. In this framework, the epidemiological model is used for understanding the spread of infectious diseases within a population, the SIR model being a fundamental one [18]. Given its simplicity, the SIR model has been adapted and extended in various ways to better capture the complexity of real-world disease transmission. For instance, the SEIR model adds an ‘exposed’ category to account for incubation periods, offering insights into COVID-19 and Ebola outbreaks [19,20]. Similarly, the SEI–SEIR model adapts to vector-borne diseases like dengue, illustrating the intricate interplay between human and mosquito populations [21]. The transmission cycle of WNV, involving multiple hosts and vectors—birds, mosquitoes and humans—necessitates a more complex modelling approach. A specialized compartmental model, denoted as $S_{b}E_{b}I_{b}R_{b}–S_{m}E_{m}I_{m}–S_{h}E_{h}I_{h}R_{h}$, is proposed to capture the unique epidemiological characteristics of WNV in [22]. The data assimilation method is used to integrate new surveillance data into the epidemiological model to update predictions and reduce uncertainties. Techniques such as the ensemble Kalman filter (EnKF) [21,23] and particle filter [24,25] are commonly used for this effort. The EnKF, by updating predictions with each new piece of information, significantly refines the accuracy of short-term forecasts and contributes to a more comprehensive understanding of disease transmission dynamics.

This study enhances the current methodology for real-time prediction of WNV. We first introduce an adapted compartment model that describes the transmission dynamics of WNV among birds, mosquitoes and humans, incorporating both the infected asymptomatic human population and the impact of weather factors such as temperature and precipitation on disease spread. We further assimilate reported human WNV cases in Colorado from June to December in 2023 into our model using the EnKF for robust data assimilation. This integration enables us to generate weekly forecasts of human WNV cases throughout the 2023 season (June to December). Such forecasts are invaluable for public health planning and intervention strategies. Moreover, our forecasting system estimates the epidemic dynamics of each involved population and calculates infection rates, providing critical insights like the estimated numbers of asymptomatic individuals and infectious mosquitoes. The precision of our forecasts is rigorously evaluated using the absolute root mean square error (ARMS) of the average forecasts and the logarithmic score (LS) of the forecasted new cases. By conducting a comparative analysis with a null negative binomial (NB) model and a model that omits weather variables, we demonstrate that incorporating temperature and precipitation enhances the accuracy of our forecasts. This finding underscores the crucial role of environmental factors in predicting WNV transmission and highlights the potential for improved public health responses through the integration of weather data into epidemiological modelling.

## 2. Material and methods

### 2.1. Data

The data utilized in our analysis are sourced from the following references: for Colorado WNV cases, we relied on data provided by the Colorado Department of Public Health and Environment, available at [26], and the CDC, available at [27]; for United States weather data, we accessed comprehensive weather information from [28]. These reliable sources ensure the accuracy and credibility of the data used in our analysis.

### 2.2. Negative binomial model

The NB model extends the Poisson regression by introducing a dispersion parameter that allows for greater variability in the data. The predicted mean number of cases, μ, is modelled as a function of covariates through a log link function:


(2.1) $$\log(\mu )=\beta_{0}+\beta_{1}X_{1}+\beta_{2}X_{2}+\cdots +\beta_{n}X_{n},$$

where $X_{i}$ are the predictor variables (e.g. time, environmental factors) and $\beta_{i}$ are the coefficients to be estimated.

In this study, the NB model is used as a baseline prediction of WNV cases in 2023, based on historical data from 2018 to 2022. The predictions generated by the NB model are then compared to the actual reported cases in 2023. The LS is used to evaluate the model’s predictive accuracy, which aligns with the scoring approach employed by the CDC [8]. This baseline serves as an important reference point, ensuring that any improvements in forecasting performance are measurable and grounded in a standard approach that has been validated in public health studies.

### 2.3. Disease model

The primary transmission cycle of WNV is between birds and mosquitoes. Many bird species are reservoir hosts for the virus. When a mosquito feeds on an infected bird, it can acquire the virus. Humans, along with other mammals like horses, are considered incidental hosts. When an infected mosquito bites a human, the virus can be transmitted. Modelling of WNV is complicated by a variety of factors: WNV has been found in 138 bird species within the United States, with susceptibility, transmissibility and infectious period varying between species; multiple mosquito vectors may be responsible for transmission. A compartment model involving five species (sparrows, crows, ornithophilic mosquitoes, generalist mosquitoes and humans) is proposed in [22]. In this model, sparrow and crow populations are categorized into susceptible (S), exposed (E), infectious (I) and recovered/removed (R) compartments; mosquito populations are divided into S, E and I compartments and the human population is divided into S, E, I and R compartments. Given the complexity of modelling interactions among 18 compartments across these five populations, the task of parametrization presents significant challenges, especially due to the difficulty in collecting data on two bird species (sparrows and crows) and the two mosquito species. Given our objective to forecast human cases and understand the epidemic dynamics within the human population, we simplify the model by consolidating the bird species into a single group and the mosquito vectors into another. Since infected humans do not transmit the virus to each other or mosquitoes, the compartment designated for infectious humans ($I_{H}$) is unnecessary and thus eliminated. It is noteworthy that approximately 80% of individuals infected with WNV remain asymptomatic [29]. Therefore, we introduce a compartment $A_{H}$ to represent the asymptomatic population and $C_{H}$ to represent the confirmed human population, refining the model to more accurately reflect the observed disease dynamics.

Transitions between compartments of the refined model are described by the following differential equations:


(2.2) $$\begin{array}{lll} {\bar{S}}_{B}^{\prime }(t) & = & v_{B}-\beta_{MB}{\bar{I}}_{M}{\bar{S}}_{B}-\mu_{B}{\bar{S}}_{B},\\ {\bar{E}}_{B}^{\prime }(t) & = & \beta_{MB}{\bar{I}}_{M}{\bar{S}}_{B}-(\delta_{B}+\mu_{B}){\bar{E}}_{B},\\ {\bar{I}}_{B}^{\prime }(t) & = & \delta_{B}{\bar{E}}_{B}-(\gamma_{B}+\mu_{B}){\bar{I}}_{B},\\ {\bar{R}}_{B}^{\prime }(t) & = & \gamma_{B}{\bar{I}}_{B}-\mu_{B}{\bar{R}}_{B},\\ {\bar{S}}_{M}^{\prime }(t) & = & v_{M}-\beta_{BM}{\bar{I}}_{B}{\bar{S}}_{M}-\mu_{M}{\bar{S}}_{M},\\ {\bar{E}}_{M}^{\prime }(t) & = & \beta_{BM}{\bar{I}}_{B}{\bar{S}}_{M}-(\delta_{M}+\mu_{M}){\bar{E}}_{M},\\ {\bar{I}}_{M}^{\prime }(t) & = & \delta_{M}{\bar{E}}_{M}-\mu_{M}{\bar{I}}_{M},\\ {\bar{S}}_{H}^{\prime }(t) & = & v_{H}-\beta_{MH}{\bar{S}}_{H}{\bar{I}}_{M}-\mu_{H}{\bar{S}}_{H},\\ {\bar{E}}_{H}^{\prime }(t) & = & \beta_{MH}{\bar{S}}_{H}{\bar{I}}_{M}-(\delta_{H}+\mu_{H}){\bar{E}}_{H},\\ {\bar{A}}_{H}^{\prime }(t) & = & \alpha \delta_{H}{\bar{E}}_{H}-\mu_{H}{\bar{A}}_{H},\\ {\bar{C}}_{H}^{\prime }(t) & = & (1-\alpha )\delta_{H}{\bar{E}}_{H}-\mu_{H}{\bar{C}}_{H}, \end{array}$$

where v and μ denote the birth rate and death rate, β denotes the infection rate, δ and γ represent the transition rate from state E to I and from I to R, α denotes the fraction of the infected asymptomatic human population, and the subscripts *B*, *M* and *H* refer to birds, mosquitoes and humans.

The model parameter definition, value and reference are summarized in table 1. U(a,b) means the parameter satisfies uniform distribution within a range from a to b and N(a,b) means the parameter satisfies normal distribution with the mean of a and the standard deviation of b.

**Table 1.** Model parameter definition, value and reference.

symbol	definition	value/distribution	reference
$v_{B}$	birth rate of birds	0.015 d^−1^	[30]
$\mu_{B}$	natural death rate of birds	U(0.0010.002) d^−1^	[31]
$\beta_{M\,B}$	infection rate of birds	estimated	none
$1/\delta_{B}$	incubation period of birds	*N*(4.5,0.5) days	[32]
$\gamma_{B}$	removing rate of infected birds	*N*(0.72,0.1) d^−1^	[32]
$v_{M}$	birth rate of mosquitoes	equation (2.3)	[30,33]
$\mu_{M}$	natural death rate of mosquitoes	equation (2.3)	[33,34]
$\beta_{B\,M}$	infection rate of mosquitoes	$\beta_{M\,B}$	assumed
$1/\delta_{M}$	incubation period of mosquitoes	*N*(9,0.3) days	[30]
$v_{H}$	birth rate of humans	0	assumed
$\mu_{H}$	natural death rate of humans	0	assumed
$\beta_{M\,H}$	infection rate of humans	estimated	none
$1/\delta_{H}$	incubation period of humans	*N*(4,1) days	[30]
α	asymptomatic fraction of infected humans	*N*(0.8,0.03)	[29]

The time-varying infection rates $\beta_{MB}$, $\beta_{BM}$ and $\beta_{MH}$ are estimated by EnKF. We assume $\beta_{MB}=\beta_{BM}$ because the estimated probability of the virus transmission to birds and that to mosquitoes is very close in previous studies [30,35,36].

For mosquitoes, both the birth and death rates are influenced by temperature and precipitation, with the magnitude of the birth rate established at 0.15 per day and the natural mortality rate for mosquitoes varying between 0.02 and 0.07 per day [30]. The birth rate of mosquitoes shows a nearly linear increase with temperature within the range of 16 to 31°C, while their mortality rate tends to decrease as temperatures rise within this same range [33]; we assume that precipitation also influences the mosquito recruitment rate in a linear fashion. Since the highest average temperature in Colorado in 2023 did not go above 31°C, we proposed the following model (2.3) without considering the situation above 31°C:


(2.3) $$\begin{array}{lll} v_{M}(T,P) & = & \left\{ \begin{array}{ll} 0.15\times 0.5\times (\frac{T-16}{T_{max}-16}+\frac{P}{P_{max}}) & 16<T<31\\ 0.01 & \text{otherwise}, \end{array}\right.\\ \mu_{M}(T) & = & \left\{ \begin{array}{ll} -\frac{T}{300}+0.12 & 16<T<31\\ 0.07 & \text{otherwise}. \end{array}\right. \end{array}$$

To isolate the impact of the weather factors on prediction accuracy, we develop a weather-neglected model that utilizes independent mosquito birth and death rates ($v_{M}∼N(0.15,0.01),\;\mu_{M}∼N(0.07,0.01)$) while keeping the other parameters unchanged.

### 2.4. Forecast model

To accurately estimate the unknown parameters and state variables, we employ the EnKF, a proficient data assimilation technique grounded in Bayesian statistics. Specifically, it leverages Bayes’ rule to iteratively update the system’s state estimate at discrete time intervals by integrating recent observations with *a priori* state estimates, thereby refining our understanding of the system’s current state.

The EnKF maintains an ensemble of state variables $X_{t}$ and time-varying parameters $\Phi_{t}=[\beta_{MH},\beta_{BM}{]}^{⊺}$, represented together in a matrix $A_{t}\in {\mathbb{R}}^{13\times N}$, where N is the ensemble size:


(2.4) $$\begin{array}{r} A_{t}=\left[\begin{array}{cccc} X_{t}^{1} & X_{t}^{2} & \cdots & X_{t}^{N}\\ \Phi_{t}^{1} & \Phi_{t}^{2} & \cdots & \Phi_{t}^{N} \end{array}\right]. \end{array}$$

At each time step, the EnKF predicts the prior, or the expected state of the system, using the model simulations


(2.5) $$A_{t+1}^{-}=F(A_{t})=\left\{ \begin{array}{l} f(X_{t}),\\ g(\Phi_{t}), \end{array}\right.\;+q_{t},$$

where $f(X_{t})$ and $g(\Phi_{t})$ denote the differential equations for the states and parameters, and $q_{t}$ represents the model noise. Thus, the ensemble covariance for the prior is


(2.6) $$\sigma_{t}^{\text{pri}}=\frac{1}{N-1}\;(A_{t}^{-}-\bar{A_{t}^{-}})\;(A_{t}^{-}-\bar{A_{t}^{-}}{)}^{⊺},$$

with $\bar{A_{t}^{-}}$ being the mean of the ensemble members.

The EnKF assimilates new observations $y_{t}$ into the ensemble $\hat{Y_{t}}=y_{t}+\epsilon_{t}$, where $\epsilon_{t}$ is the observation perturbation vector. The assimilation step compares the predicted states of the ensemble with observed data, adjusting the estimated state and parameters to better fit the observations:


(2.7) $$A_{t}=A_{t}^{-}+K_{t}\;(\hat{Y_{t}}-H_{t}A_{t}^{-}),$$

where $K_{t}=\sigma_{t}^{\text{pri}}H_{t}^{⊺}(H_{t}\sigma_{t}^{\text{pri}}H_{t}^{⊺}+\sigma_{t}^{\text{obs}}{)}^{-1}$ is the Kalman gain, $H_{t}$ is the observation operator and $\sigma_{t}^{\text{obs}}\;=\;\epsilon_{t}\epsilon_{t}^{⊺}\;/\;N-1$ represents the observation error covariance.

By perturbing the updated estimated state and parameters, the EnKF generates a new ensemble of model simulations for the next time step.

In our study, we ran a 200-member ensemble simulation of the SEIR–SEI–SEAC compartmental model with human WNV case data using the EnKF. The system integrates the modelled state space, comprising 11 disease state variables and two parameters, with weekly observations of human WNV cases. The EnKF algorithm assimilates new observations to update the model’s observed state variables whenever they become available. It also adjusts the model’s unobserved state variables and parameters using cross-ensemble co-variability, advancing the model to the next observation period with updated (posterior) state variables and parameters. This iterative optimization process aligns the ensemble of model simulations more closely with current local outbreak dynamics.

## 3. Results

### 3.1. Correlation of human West Nile virus cases with local temperature and precipitation

**Figure 1.**
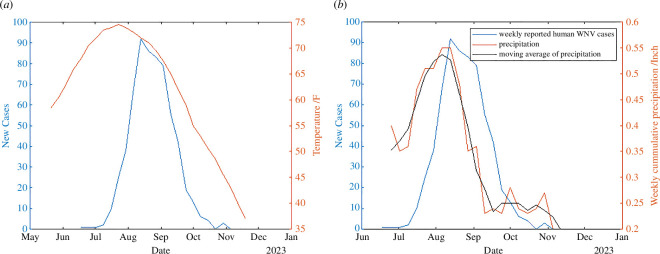
Weekly human WNV cases, average temperature and weekly precipitation. (*a*) Cases and average temperature. (*b*) Cases and weekly precipitation.

Figure 1 shows the weekly reported human WNV cases, the weekly average temperature and the weekly cumulative precipitation. From the figures, we can observe that the weekly reported cases are lag-correlated with the temperature and the precipitation. The correlation coefficient between WNV cases and average temperature is 0.69 with a lag of three weeks. When calculating the coefficient between WNV cases with precipitation, we use the moving average with window size three of the precipitation to reduce the noise of the observation. The correlation coefficient between WNV cases and the moving average of precipitation is 0.57 with a one-week lag. This finding indicates that the temperature and precipitation have the potential to inform reliable prediction of human WNV cases.

Our analysis reveals a significant correlation between the reported weekly human WNV cases and the weather variables (temperature and precipitation). Given that our SEIR–SEI–SEAC model includes 11 states, we investigate the correlation coefficients of parameters across higher orders. Interestingly, we observe that the correlation intensifies with the increasing powers of weather variables. Specifically, the temperature raised to the ninth power exhibits the highest correlation coefficient of 0.88 with the human cases, whereas for precipitation, a power of six yields a correlation coefficient of 0.65, as detailed in table 2. This suggests that the relationship between weather factors and WNV transmission may be more complex than linear associations, and points to the need for high-dimensional systems to depict the intricate dynamics between weather factors and WNV transmission. Our SEIR–SEI–SEAC model incorporates 11 states, allowing for the exploration of parameters with high orders.

**Table 2.** The correlation coefficients r between human WNV cases and higher orders i of weather variables (temperature T and precipitation P).

i	2	3	4	5	6	7	8	9	10
$r_{T^{i}}$	0.73	0.77	0.79	0.82	0.84	0.85	0.87	0.88	0.88
$r_{P^{i}}$	0.60	0.62	0.63	0.64	0.65	0.65	0.65	0.65	0.65

### 3.2. Real-time forecast

**Figure 2.**
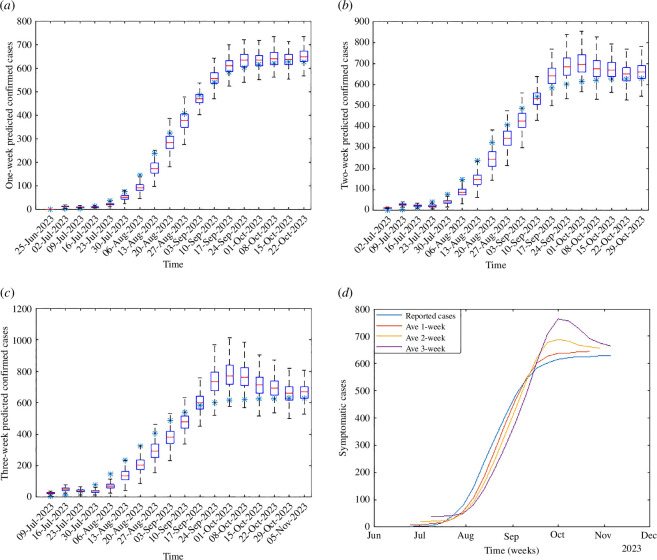
Real-time prediction of the cumulative WNV cases in Colorado. (*a*) One-week forecast. (*b*) Two-week forecasts. (*c*) Three-week forecasts. (*d*) Average forecasts.

The disease model SEIR–SIE–SEAC is applied to the EnKF to generate real-time forecasting of human WNV cases in Colorado, USA, for the year 2023. The forecasting process starts at the onset of the outbreak on 4 June, utilizing the cumulative confirmed human WNV cases $C_{H}$ as the observation. The data, in combination with average temperature and weekly cumulative precipitation, are assimilated weekly into the EnKF. As a result, the system is able to generate forecasts for the forthcoming three weeks at each assimilation point. This approach yields three sets of forecast leads during the simulation (where the one-week lead forecasts pertain to the following week, the two-week lead forecasts to the week after next and so on). The simulation process was iterated 500 times to ensure robustness. The prediction results of the cumulative confirmed cases are shown in figure 2, where figure 2*a*–*c* delineates the distribution of the prediction across the three weeks. In these figures, observed case counts are denoted by stars, whereas the forecast distributions are illustrated with box plots: the extremities of the distributions are marked by black lines, the interquartile ranges by blue boxes and the medians by red lines. Figure 2*d* presents the average forecast trajectories. It is observed that the actual case numbers predominantly fell within the forecasted range, with shorter lead times yielding more precise predictions. The average forecasts capture the general trend of the epidemic’s growth.

**Figure 3.**
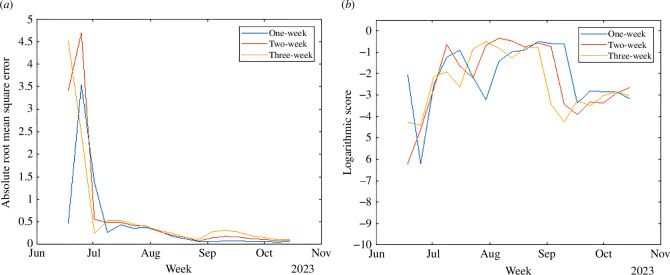
Accuracy evaluation. (*a*) Absolute root mean square error. (*b*) Logarithmic score.

The effectiveness of the forecasting model is assessed by the ARMSE and the LS. The ARMSE metric measures the average magnitude of the prediction errors, providing a clear indication of how far our predictions deviate from the observed values. It is particularly useful for understanding the overall accuracy of our forecast in numerical terms. On the other hand, the LS evaluates the probability of our predictions falling within the same range (bin) as the observed outcomes. This metric is essential for assessing the reliability of our probabilistic forecasts and ensuring that our model’s uncertainty is appropriately calibrated. To compute the LS, we first deduce the probability distribution of new case occurrences from the cumulative case predictions. Then, we classify case counts into predetermined bins (0, 1−5, 6−10, …, 46−50, 51−100, 101−150, 151−200 and >200), aligning with the categorization scheme utilized in the CDC challenge [12]. The outcomes are depicted in figure 3, where the ARMSE values for each forecast lead after 9 July (the fourth data point) are maintained below 0.5 as shown in figure 3*a*, signifying a high level of precision in the model’s predictions relative to the observed data; and the LS remains robust, generally higher than −3 during the epidemic’s peak phases. This indicates that after an initial period of model adjustment and alignment with the actual epidemiological conditions, the forecasting system demonstrates a remarkable capability to generate real-time predictions with significant accuracy.

### 3.3. Accuracy evaluation of the negative binomial model

**Figure 4.**
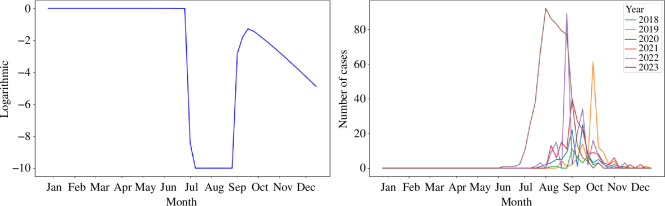
Logarithmic score of the NB model and the historical case data. (*a*) Logarithmic score. (*b*) Weekly historical cases.

The NB model produces a probability distribution across predefined bins (0, 1−5, 6−10, …, 46−50, 51−100, 101−150, 151−200 and >200) for each week in 2023. These results are evaluated using the LS, as shown in figure 4*a*. From the figure, we observe that the model’s performance between July and December is poor, with LS consistently below −10. This period coincides with the peak of the WNV outbreak, suggesting that the model struggles to accurately predict cases during the most critical phase of the outbreak. The average LS of the NB model after July is −5.5.

In figure 4*b*, we plot the weekly WNV cases from 2018 to 2023 and note that the 2023 outbreak started earlier and had a larger magnitude compared to the previous five years. The time period when the NB model achieves higher scores corresponds to weeks when the 2023 case counts are similar to those in prior years. Overall, while the NB model captures the general trends in historical outbreak data, it is unable to account for the irregularities and higher-than-expected case counts in 2023.

### 3.4. Accuracy evaluation of the weather-neglected model

**Figure 5.**
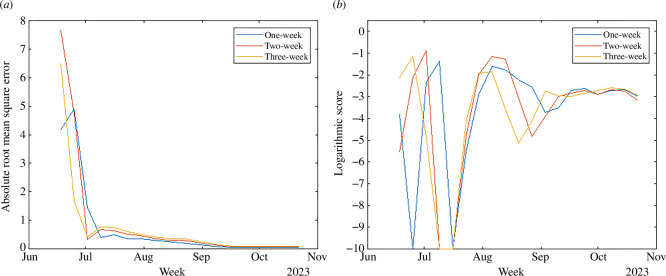
Accuracy evaluation of the weather-neglected model. (*a*) Absolute root mean square error. (*b*) Logarithmic score.

Figure 5 presents the ARMSE and LS of the forecasts from the weather-neglected model. Comparing with figure 3, we observe that the model incorporating weather variables consistently outperforms the weather-neglected model. Specifically, the average ARMSE for the weather-neglected model are 0.19, 0.27 and 0.31 for successive leads after 9 July, whereas the weather-incorporated model achieves improved averages of 0.17, 0.23 and 0.28, respectively; similarly, the average LS for the weather-neglected model is −3.2, −3.8 and −3.9 for each forecast lead, in contrast to the more favourable scores of −1.8, −1.8 and −2.2 achieved with the integration of weather variables. Overall, there is a 1.6 to 2.2 point improvement in average LS for the weather-neglected model over the NB model, and a 1.4 to 1.7 point improvement for the weather-incorporated model over the weather-neglected model. These findings demonstrate that a real-time forecasting system is crucial when the outbreak’s behaviour—such as timing or case count—differs significantly from that of previous years. Moreover, our results underscore the substantial benefits of incorporating weather variables into epidemic forecasting models, highlighting their critical role in enhancing forecast accuracy for human WNV cases.

### 3.5. Estimated epidemic dynamics

**Figure 6.**
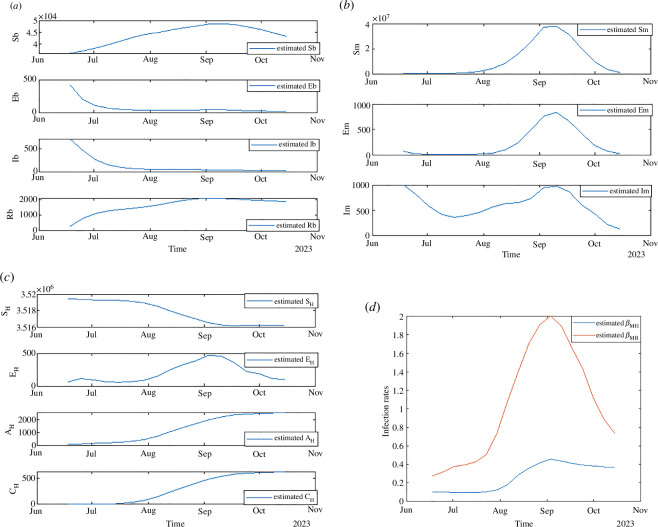
Estimated epidemic dynamics. (*a*) Birds, (*b*) mosquitoes, (*c*) humans and (*d*) infection rates.

Our system can also estimate both unobserved states and unknown parameters by assimilating limited observational data. Figure 6 shows the estimated epidemic dynamics across different species, representing the progression of the outbreak. To ensure the model reflects an outbreak scenario, we initiate it with a substantially large number of initially infected birds and mosquitoes. The EnKF will align the model’s state trajectories close to actual conditions as the outbreak starts. From figure 6*a*,*b*, we observe that the population of infectious mosquitoes greatly exceeds that of infectious birds throughout the epidemic, which can be attributed to the longer infectious period of mosquitoes, who remain carriers of the virus until their death, while birds exhibit a high removal rate due to recovery or death, which significantly shortens the duration the virus persists within their system. As shown in figure 6*d*, the infection rate between mosquitoes and birds surpasses that from mosquitoes to humans, indicating a higher susceptibility of birds to the WNV. These observations highlight the critical role of mosquitoes in spreading the virus and birds as essential reservoirs for the virus and further emphasize the need for targeted vector control strategies to mitigate the outbreak. In figure 6*c*, the reported number of human cases in Colorado reached 621 by 8 October. However, our model estimates indicate the presence of approximately 2518 asymptomatic individuals, alongside 100 exposed individuals who may still develop symptoms.

## 4. Conclusion

The forecasting target of this study is the weekly cumulative cases. Forecasting weekly cumulative cases helps track the trajectory of the outbreak, providing crucial insights for understanding the immediate burden on healthcare systems. This enables public health officials to allocate resources effectively, ensuring that hospitals are prepared for potential surges with adequate medical supplies, personnel and facilities. Additionally, we also deliver estimates for the number of exposed individuals; infection rates between birds, mosquitoes and humans; and the asymptomatic human population, which are made with careful consideration of public health needs. Estimating the exposed population provides insights into the potential future spread of the virus, which is vital for planning targeted interventions such as public awareness campaigns and vector control measures in areas with high exposure risk. Understanding the infection rates between birds, mosquitoes and humans is essential for implementing specific vector control strategies, such as insecticide spraying and habitat reduction, to interrupt the transmission cycle. Estimating the asymptomatic population is critical for assessing the true extent of the outbreak, helping in planning broader testing strategies and more accurate risk communication. A three-week forecasting horizon was chosen to balance the need for actionable lead time with the accuracy of predictions. This period is deemed sufficiently early for several meaningful public health responses, such as resource allocation, public awareness and communication and decision-making (e.g. park closures and travel advisories). We are actively working on extending the prediction lead time while ensuring that the accuracy of the predictions is maintained. State-level results can be used for coordinating large-scale public health responses. While the state-level forecasts are presented in this study, our system is also capable of delivering smaller-scale predictions and estimations, such as at the county level, provided the relevant data (cases, local temperature and precipitation) are available. This flexibility allows for more localized public health interventions, enhancing the overall effectiveness of outbreak management strategies.

While our model shows strong predictive capabilities for the WNV outbreak in Colorado during the 2023 season, it is important to acknowledge its limitation of being designed for a single location and single season. This focus allows for detailed and accurate predictions within this specific context but may not be directly applicable to other regions or periods due to varying transmission dynamics influenced by differing weather conditions, vector behaviours and public health interventions. Caution should be exercised when generalizing these findings, and future research should aim to adapt and validate the model across multiple locations and seasons to assess its robustness and enhance its applicability for broader public health applications.

The forecasting model could also be improved by incorporating the effects of human interventions and public health measures implemented during outbreaks. Future work could focus on integrating these human intervention factors into the forecasting model to enhance its predictive accuracy and usefulness for public health planning. In addition, given the diversity of host species, there is a clear need for more detailed data collection. Future work should aim to gather comprehensive information on the transmission dynamics across different host species and integrate these data into the model, potentially improving the accuracy and specificity of forecasts.

## Data Availability

Data and relevant code for this research work are stored in GitHub [37] and have been archived within the Zenodo repository [38].
